# Hantavirus Research in Finland

**DOI:** 10.3390/v16101591

**Published:** 2024-10-10

**Authors:** Jukka Mustonen, Tomas Strandin, Johanna Tietäväinen, Ilkka Pörsti, Satu Mäkelä, Antti Vaheri

**Affiliations:** 1 Faculty of Medicine and Health Technology, Tampere University, 33014 Tampere, Finland; johanna.tietavainen@pirha.fi (J.T.); ilkka.porsti@tuni.fi (I.P.); satu.m.makela@pirha.fi (S.M.); 2 Department of Internal Medicine, Tampere University Hospital, 33520 Tampere, Finland; 3 Department of Virology, Medicum, University of Helsinki, 00290 Helsinki, Finland; tomas.strandin@helsinki.fi (T.S.); antti.vaheri@helsinki.fi (A.V.)

## 1. Introduction

The articles in this Special Issue, “Hantavirus Research in Finland”, were published between 2021 and 2022. In this closing editorial, we comment on some of these papers, with a primary focus on future research directions that should be considered. We also describe the main findings of other recent reports on hantavirus research published in Finland in the past few years.

## 2. Immune Response and Pathogenesis

Several aspects of the immune response and pathogenesis of Puumala virus (PUUV) infection have been studied by Finnish scientists [1]. In this Special Issue, Iheozor-Ejiofor et al. reported that neutralizing antibody titers are not associated with the clinical severity of acute kidney injury (AKI) in PUUV infection [2]. It has previously been reported that a strong neutralizing antibody response in patients with hantavirus cardiopulmonary syndrome (HCPS) caused by Sin Nombre virus (SNV) may predict effective clearance of, and recovery from, SNV infection, suggesting that passive immunotherapy could be useful in HCPS [3]. More research is needed to investigate the potential of these antibodies as therapy for hantavirus infections.

Proteinuria is present in most patients with PUUV-induced hemorrhagic fever with renal syndrome (HFRS). In this Special Issue, Cabrera et al. showed that the local activation of heparinase (HPSE) in the kidneys of the patients may disrupt the endothelial glycocalyx, leading to increased protein leakage through the glomerular basement membrane (GBM), resulting in proteinuria [4]. Further studies are needed to explore the potential use of HPSE inhibitors as therapeutic agents in acute PUUV infection.

In 2021, our group published three articles, not included in this Special Issue, about the immune response in PUUV infection. In the first of them, it was reported that tetanus- and pertussis-specific IgG concentrations increase during acute infection [5]. Interestingly, the increase in tetanus-specific IgG persisted for a year after PUUV infection, while pertussis-specific IgG declined rapidly, highlighting the difference in IgG kinetics observed after vaccination against tetanus and pertussis [5].

The second study in 2021 demonstrated that hantaviruses directly activate B cells and that the ensuing intense production of polyclonal immunoglobulins and free light chains may contribute to hantavirus-associated pathological findings [6].

The main conclusion of the third study was that during severe PUUV-induced hemorrhagic fever with renal syndrome (HFRS), monocytes are activated upon exposure to PUUV in the blood, stimulating endothelial adhesion and subsequent redistribution from circulation to the kidneys [7].

In a previous report in 2018, we showed that PUUV-induced hantavirus infection is characterized by strong neutrophil activation [8]. In a recent study, an identification and characterization of different neutrophil subsets in the circulation of PUUV-HFRS patients were performed [9]. Low-density granulocytes (LDGs) were increased, and interestingly, both the frequency of LDGs and the presence of a “left shift” in blood were associated with the extent of thrombocytopenia, one of the hallmarks of HFRS. The conclusion was that maturing neutrophils may play a role in disease pathogenesis, a finding that represents an interesting area for further exploration [9].

In another recent study performed in collaboration with Finnish and Swedish scientists, a detailed characterization of peripheral blood innate lymphoid cells (ILCs) and natural killer (NK) cells was performed [10]. This study was the first comprehensive characterization of total circulating ILCs in hantavirus-infected patients. The results showed that ILCs are activated in HFRS patients, and the findings suggested that classical antiviral type I interferons (IFNs) are involved in shaping ILC functions. Further studies of ILCs in tissue samples, such as those from lungs and intestines, could provide important insights into possible ILC tissue infiltration and local ILC responses [10].

## 3. Clinical Course

PUUV infections show extensive variation in clinical outcomes, ranging from asymptomatic seroconversion to a fatal outcome. A novel finding was reported by Tietäväinen et al. in this Special Issue. Specifically, plasma glucose levels during the acute phase of the disease are associated with the severity of capillary leakage, thrombocytopenia, inflammation, and AKI [11].

As discussed, a higher plasma glucose level may simply be a sign of more severe disease. However, it is also possible that glucose influences the pathophysiological process of PUUV infection by damaging the vascular endothelium through several mechanisms [11]. Therefore, further studies are needed on this interesting topic.

A recent review by Vaheri et al. addressed the great variation in the clinical course of PUUV infection, posing the question, “Why this variation?” [12]. It was suggested that multiple host genetic factors may play a role. Immunogenetic investigations have mainly focused on the human leukocyte antigen (HLA) and genes that encode molecules associated with this complex, such as the C4A component of the complement system. As reported in this Special Issue by Tietäväinen et al., ABO and rhesus blood groups do not have any major influence on the susceptibility to or severity of PUUV infection [13].

Host immune and inflammatory mechanisms are likely important in the pathogenesis leading to clinical symptoms of varying severity. There seem to be no differences in the virulence of PUUV strains infecting humans, although this has not been thoroughly studied thus far [12].

One hallmark of hantavirus infections is increased capillary permeability, which is likely a consequence of the complex interplay between viral infection and host immune responses. In a recent study, magnetic resonance imaging (MRI) was used to evaluate the presence of intraperitoneal, retroperitoneal, and pleural fluid among 27 patients with acute PUUV infection [14]. Fluid collections were found in all cases. The amount of intraperitoneal fluid correlated positively with the degree of inflammation, as measured by plasma levels of C-reactive protein (CRP). Interestingly, the amount of both intra- and retroperitoneal fluids had an inverse correlation with the severity of AKI, as measured by serum creatinine [14].

Acute back pain was present in 67% of patients [14]. It was associated with the severity of AKI but not with the amount of fluid collections. The findings suggest that the pathogenesis of AKI and increased capillary leakage proceed independently of each other in PUUV infection. More prominent intra- and retroperitoneal fluid collections may even appear as a protective factor against the development of more severe AKI [14]. These novel findings about vascular leakage in PUUV infection warrant further investigation to be confirmed.

Several long-term consequences of PUUV infection have been reported in the literature [15]. One consequence is lymphoma. Studies conducted among Swedish [16] and Korean [17] populations have shown an increased risk of lymphoid malignancies following PUUV and Hantaan (HNTV) virus infections, respectively. A recent study documented that PUUV infection is also associated with lymphoid malignancies in the Finnish population [18]. The risk of lymphoma increased relatively soon after PUUV infection and remained elevated for up to five years. As concluded, further research is needed to understand the biological mechanisms underlying this association [18].

## 4. Epidemiology and Ecology

A recent literature review reported on hantavirus infections described among military forces [19]. In 1942, a large epidemic caused by PUUV occurred during World War II among German and Finnish soldiers in Northern Finland, affecting more than 1000 patients. During the Korean War, from 1951 to 1954, about 3200 cases occurred among United Nations soldiers in an epidemic caused by HNTV. During the Balkan War, from 1991 to 1995, many soldiers fell ill due to infections caused by PUUV and the Dobrava virus (DOBV).

In addition, several reports have documented hantavirus infections among military personnel not in active war situations. These infections have occurred among U.S. soldiers stationed in South Korea, Germany, Bosnia, and Kosovo. It is quite possible that hantavirus infections may also emerge among military troops and civilians during the ongoing war in Ukraine. Notably, it is known that pathogenic hantaviruses are present in some common rodent species in Ukraine [19].

An ecological study from Finland investigated how factors affecting pathogen transmission in wildlife hosts can be important for predicting human disease outbreaks [20]. The results suggested that certain predator species (e.g., red foxes and weasels), a high proportion of young bank voles (*Myodes glareolus*), and a diverse rodent community may reduce the risk of PUUV for humans by negatively impacting the abundance of infected bank voles [20].

## 5. Treatment

The successful use of icatibant, a drug licensed for the treatment of acute episodes of hereditary angioedema (HAE), has been reported in two extremely severe cases of PUUV infection [1]. Icatibant acts as a selective antagonist of the bradykinin (BK) type 2 receptor, reducing vascular permeability and inhibiting vasodilation.

Recently, it was shown that adding this drug to standard care was safe and improved outcomes for both COVID-19 pneumonia and mortality [21]. There are many similarities in the pathogenesis of hantavirus infections and COVID-19. Therefore, icatibant should be studied for the treatment of more severe forms of hantavirus infections than PUUV infection, including cases caused by DOBV in Europe and HNTV in Asia, and especially in patients with hantavirus cardiopulmonary syndrome (HCPS) caused by Andes and related viruses in North and South America [22].

## 6. Conclusions

The scientific research among hantavirus infections started in Finland more than 50 years ago [9]. A recent bibliometric analysis of hantavirus research included 4408 studies published from 1980 to 2020 [23]. The highest number of studies came from the USA, with Finland ranking fourth. The list of institutions was globally led by the University of Helsinki, and Antti Vaheri was identified as the most influential author [23].

Active research on this topic continues in Finland. Close collaboration between virologists at the University of Helsinki and clinicians at Tampere University and Tampere University Hospital has resulted in numerous reports from Finland over the past several decades. These groups have a fruitful collaboration with zoologists in Finland and scientists at Karolinska Institute in Stockholm, Sweden [7,10].
